# Association Between Preoperative Blood Glucose Level and Hospital Length of Stay in Patients With Kidney Stones Undergoing Percutaneous Nephrolithotomy

**DOI:** 10.3389/fsurg.2021.820018

**Published:** 2022-01-20

**Authors:** Si Sun, Weipu Mao, Shuchun Tao, Lilin Wan, Xiangyu Zou, Guangyuan Zhang, Ming Chen

**Affiliations:** ^1^Department of Urology, Zhongda Hospital, Southeast University, Nanjing, China; ^2^Surgical Research Center, Institute of Urology, Southeast University Medical School, Nanjing, China; ^3^Department of Urology, Nanjing Lishui District People's Hospital, Zhongda Hospital Lishui Branch, Southeast University, Nanjing, China; ^4^School of Basic Medical Sciences, Weifang Medical University, Weifang, China

**Keywords:** kidney stones, length of stay, percutaneous nephrolithotomy, preoperative blood glucose, retrospective study

## Abstract

**Aim:**

To assess the effect of preoperative blood glucose (POBG) levels on the length of stay (LOS) in patients with kidney stones undergoing percutaneous nephrolithotomy (PCNL).

**Methods:**

We conducted a retrospective study of patients who underwent PCNL at the Zhongda Hospital of Southeast University from 2013 to 2019. The relationship between POBG level and LOS was investigated by dose-response analysis curves of restricted cubic spline function.

**Results:**

We included 310 patients and divided them into three groups (<5.04, 5.04 to <6.88, ≥6.88 mmol/L) according to the POBG levels. Patients with POBG levels ≥6.88 mmol/L (adjusted odds risk [aOR] 1.67; 95% CI 0.83–3.33) had a 67% higher risk of LOS > 2 weeks than patients with POBG levels <5.04 mmol/L. A positive dose-response analysis curve was observed between POBG and the adjusted risk of LOS >2 weeks. Similar results were observed in the subgroups analysis.

**Conclusion:**

We demonstrated that higher POBG levels were significantly associated with longer LOS in patients with kidney stones undergoing PCNL.

## Introduction

Kidney stones are a common disease of the urinary system, with a prevalence of 12% in adult men and 5% in women in Western countries, with an increasing trend in recent years (1). In China, the prevalence rate of kidney stones in adults is 5.8% (2). Percutaneous nephrolithotomy (PCNL) has become the treatment of choice for patients with larger kidney stones (>2 cm) (3). Studies have shown that obesity, diabetes, hypertension and metabolic syndrome are risk factors for stone formation (4, 5). Infection is one of the most important complications after PCNL, which may cause sepsis or even life-threatening infectious shock (6). Clinical studies have shown that preoperative hyperglycemia is an independent risk factor for the development of sepsis after PCNL (5, 7).

More recent studies indicate that there is a significant correlation between perioperative hyperglycemia and adverse clinical outcomes (8–11). The increased risk of postoperative complications in patients is related to the severity of perioperative hyperglycemia. Few studies have examined the impact of preoperative blood glucose (POBG) levels on prognosis, and data on optimal preoperative blood glucose management are also lacking (12, 13). Therefore, there is no standardized monitoring of routine POBG levels. At the same time, the latest European Society of Anesthesiology (ESA) guidelines do not recommend routine POBG assessment for patients undergoing elective non-cardiac surgery (14).

Optimizing preoperative glucose management may have prognostic value in patients undergoing elective surgery. Abdelmalak et al. (15), retrospectively analyzed clinical data from 61,536 patients undergoing elective non-cardiac surgery. They found that crude incidence of postoperative complications was significantly associated with preoperative glucose levels, with an incidence of 8–11% in patients with glucose levels of 4–6 mmol/L and 12–16% in those above seven mmol/L. Therefore, there is no standardized monitoring of routine POBG levels. At the same time, the latest European Society of Anesthesiology (ESA) guidelines do not recommend routine POBG assessment for patients undergoing elective non-cardiac surgery (14).

In the present study, we analyzed the influence of preoperative blood glucose on the length of hospital stay in patients with kidney stones, and analyzed the possible influencing factors. We found that the preoperative blood glucose level was positively correlated with the length of hospital stay. This also provides a reference for optimizing blood glucose in patients with kidney stones before surgery.

## Patients and Methods

### Patients Selection

In this study, we retrospectively collected the data of patients with kidney stones who underwent PCNL between December 2013 and December 2019 in the Department of Urology, Zhongda Hospital. After applying exclusion criteria, 310 patients were finally included. The methodology of this study followed the criteria outlined in the Helsinki Declaration (revised in 2013), and ethical approval was obtained from the Ethics Committee and Institutional Review Board for Clinical Research of Zhongda Hospital (Lot No. 2021ZDSYLL138-P01). All patients or their relatives who participated were informed and signed an informed consent form.

All patients, who underwent PCNL with POBG levels recorded within 24 h before surgery, were included in this study. The exclusion criteria were as follows: (a) Patients with malignant tumors (*n* = 7); (b) Patients with incomplete or missing follow-up data (*n* = 8); (c) Patients undergoing immunosuppressive treatment (*n* = 2); (d) PCNL for pediatrics.

Postoperative complications occurred in 68 patients, most of them were minor complication such as fever, hematuria and irritative symptoms. According to Clavien-Dindo grade (16), 13 Clavien grade III–IV complications were encountered in treating stone disease. Four patients suffered from uroseptic shock characterized by acute circulatory failure and persistent arterial hypotension unexplained by other causes (17). All the patients who suffered from uroseptic shock were diagnosed in 6 h after surgery. After 2–4 days of close monitoring and resuscitation, the patients' condition gradually stabilized. Three other patients developed postoperative renal hemorrhage, for which conservative treatment was ineffective, and were eventually treated with renal artery interventional embolization. The longest hospitalized patient was a 64-year-old male with a total hospital stay of 38 days. He developed postoperative septic shock and returned to the general ward after 2 weeks of intensive care in the ICU. He developed persistent hematuria after removal of the nephrostomy tube and was discharged in good condition after interventional embolization.

### Glucose Measurement

The major independent variable in this study was the fasting glucose level, which was measured within 2 days before surgery. In this study, plasma was collected from patients during morning fasting for blood glucose measurement. Plasma from all patients was tested on the Architect ci 16200 system (Abbott Laboratories). Thresholds were determined based on the 25th and 75th percentiles of blood glucose levels.

### Data Collection

Patients' clinical information was obtained from hospital electronic medical records. It included gender (male and female), age (<55 and ≥55 years), body mass index (BMI) (<24 and ≥24 kg/m^2^), hypertension (no and yes), diabetes (no and yes), cardiovascular disease (no and yes), smoking (no and yes), and Clavien-Dindo grade (I, II, III, and IV). The outcome of interest is the hospital's LOS. The LOS is defined as the interval between the date of discharge and the date of admission to the hospital. In China, the length of hospital stay also includes the time of preoperative examination, preoperative preparation time, and postoperative recovery time. Generally, the average length is about 2 weeks.

### Statistical Analysis

Continuous variables are expressed as mean ± standard deviation, and *t*-test for slope is used for generalized linear models. Categorical variables are expressed as *n* (%) and analyzed by the χ^2^ test. Hospital LOS was considered as a dichotomous variable ( ≤ 2 weeks or >2 weeks). The relationship between POBG and hospital LOS > 2 weeks was assessed using univariate and multivariate logistic regression. In multivariate logistic regression, we constructed three models to assess the relationship between POBG and hospital LOS and calculated the adjusted advantage ratio (aOR). In the basic model, we adjusted for age, gender, and BMI. Then, we further adjusted for hypertension, diabetes, cardiovascular disease, and smoking in the core model. Finally, in the extended model, we adjusted for white blood cells (WBC), neutrophil-to-lymphocyte ratio (NLR), and Clavien-Dindo grade.

The restricted cubic spline function is a powerful tool for describing the dose-response relationship between continuous variables and outcomes. We used the restricted cubic spline function to describe the dose-response relationship between POBG and hospital LOS >2 weeks, adjusted for variables in the extended model (18). We also estimated the aOR and 95% CI for hospital LOS >2 weeks corresponding to a specific POBG. In subgroup analyses, we performed the same analysis for subgroups with proportions > 50% to determine the relationship between POBG and hospital LOS. Studies were analyzed using SPSS software (version 24.0) and RStudio software (version 1.2.5033), and P <0.05 was considered statistically significant.

## Results

The characteristics and demographics of patients in this study were shown in Table 1. The average age of patients included in this study was 55.88 years. By trisecting POBG levels, we divided all patients into three groups: <5.04 mmol/L group, 5.04 to <6.88 mmol/L group, and ≥6.88 mmol/L group. At higher POBG levels (≥6.88 mmol/L), an increasing trend in median age and prevalence of hypertension was observed. Patients with POBG levels in the highest tertile had longer hospital durations compared to other patients. Complications distribution hinged on Clavien-Dindo grade was similar among these three groups. The preoperative length of stay and stone burden of the admitted patients also did not differ significantly among the three groups. In addition, WBC and NLR were positively correlated with POBG levels (Supplementary Figure 1).

**Table 1:** Baseline characteristics by preoperative glucose level in patients undergoing percutaneous nephrolithotomy.

		**Percutaneous nephrolithotomy (*****n*** **=** **310)**			
**Characteristic**	**All patients**	**Glucose** ** <5.04**	**Glucose 5.04 to** ** <6.88**	**Glucose** **≥6.88**	***P*** **-value**
	**No. (%)**	**No. (%)**	**No. (%)**	**No. (%)**	
Total patients	310	76 (24.5)	156 (50.3)	78 (25.2)	
Age, years	55.88 ± 11.93	51.22 ± 13.13	55.73 ± 11.18	60.72 ± 10.33	<0.001
Age categorized, years					<0.001
<55	141 (45.5)	46 (60.5)	73 (46.8)	22 (28.2)	
≥ 55	169 (54.5)	30 (39.5)	83 (53.2)	56 (71.8)	
Gender					0.196
Male	199 (64.2)	55 (72.4)	94 (60.3)	50 (64.1)	
Female	111 (35.8)	21 (27.6)	62 (39.7)	28 (35.9)	
BMI, kg/m^2^	25.25 ± 4.30	24.97 ± 4.53	25.23 ± 4.49	25.54 ± 3.70	0.416
BMI categorized, kg/m^2^					0.600
<24	120 (38.7)	33 (43.4)	57 (36.5)	30 (38.5)	
≥24	190 (61.3)	43 (56.6)	99 (63.5)	48 (61.5)	
Hypertension					0.002
Yes	120 (38.7)	19 (25.0)	60 (38.5)	41 (52.6)	
No	190 (61.3)	57 (75.0)	96 (61.5)	37 (47.4)	
Diabetes					0.713
Yes	51 (16.5)	11 (14.5)	25 (16.0)	15 (19.2)	
No	259 (83.5)	65 (85.5)	131 (84.0)	63 (80.8)	
Cardiovascular diseases					0.271
Yes	14 (4.5)	2 (2.6)	6 (3.8)	6 (7.7)	
No	296 (95.5)	74 (97.4)	150 (96.2)	72 (92.3)	
Smoking					0.593
Yes	11 (3.5)	3 (3.9)	4 (2.6)	11 (5.1)	
No	299 (96.5)	73 (96.1)	152 (97.4)	299 (94.9)	
WBC	7.00 ± 2.26	6.81 ± 2.29	7.03 ± 2.21	7.13 ± 2.34	0.391
NLR	3.09 ± 2.75	2.48 ± 1.76	3.09 ± 2.80	3.68 ± 3.29	0.007
Stone burden, cm	2.04 ± 0.43	1.99 ± 0.35	2.06 ± 0.43	2.09 ± 0.48	0.349
Complications					0.283
Grade I	32(10.3)	9(2.9)	11(3.5)	12(3.9)	
Grade II	23(7.4)	5(1.6)	7(2.3)	11(3.5)	
Grade III	9(2.9)	4(1.3)	2(0.6)	3(1.0)	
Grade IV	4(1.3)	0(0)	2(0.6)	2(0.6)	
Glucose, mmol/L	6.33 ± 2.22	4.70 ± 0.46	5.74 ± 0.63	9.10 ± 2.77	<0.001
Preoperative LOS, days	4.65 ± 1.45	4.54 ± 1.23	4.55 ± 1.53	4.96 ± 1.46	0.087
LOS, days	15.21 ± 5.31	14.34 ± 4.65	14.56 ± 4.83	17.36 ± 6.22	<0.001
LOS > 2 weeks	140 (45.2)	29 (38.2)	65 (41.7)	46 (59.0)	0.016

*Continuous variables were presented as the mean ± standard deviation and categorical variables were expressed as n (%). For categorical variables, P-values were analyzed by χ^2^ test. For continuous variables, the t-test for slope was used in generalized linear models*.

*BMI, Body mass index; LOS, Length of stay*.

**Figure 1:**
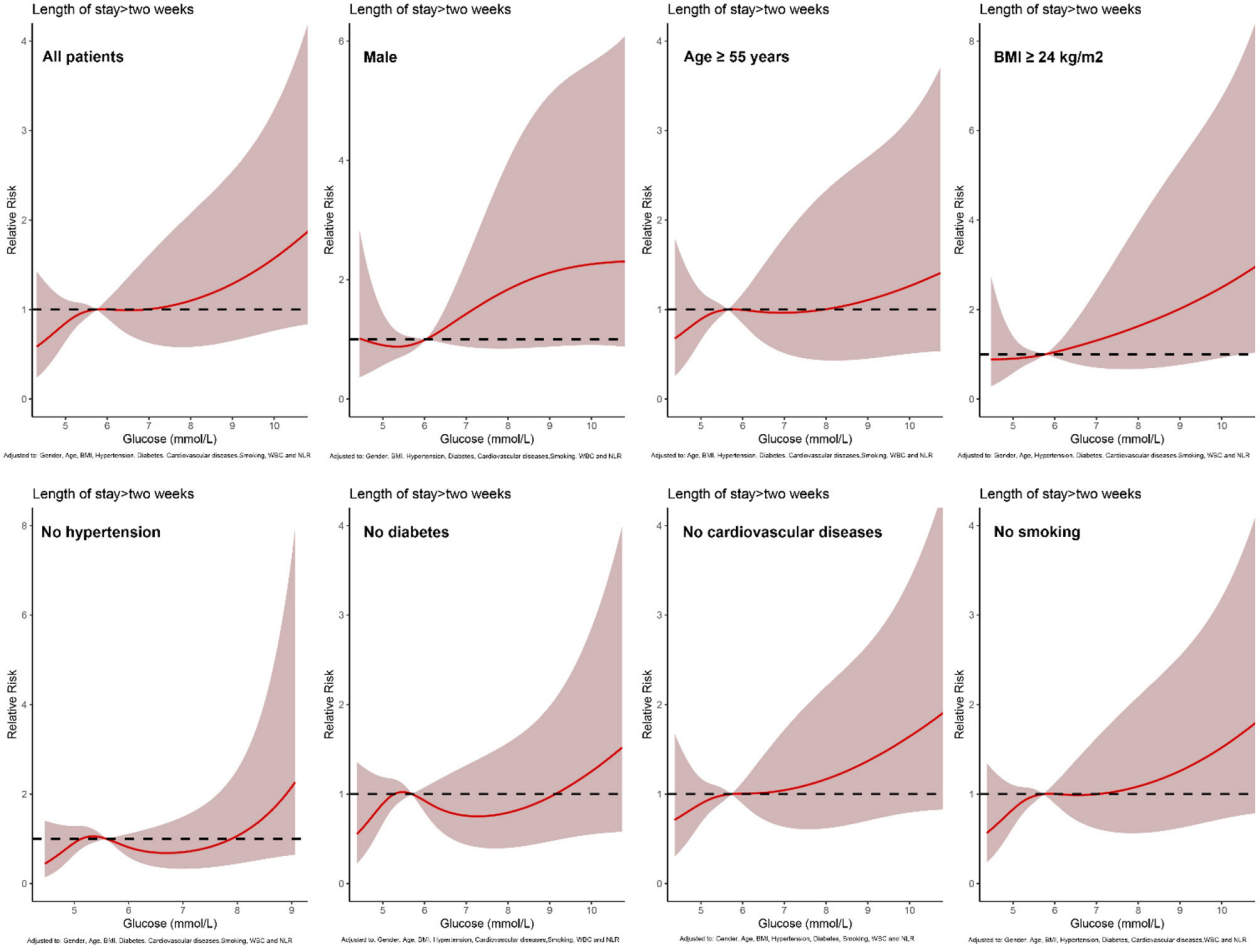
Relative risk for a hospital LOS > 2 weeks based on POBG level. The solid black lines represent aORs according to restricted cubic splines for POBG level. The shaded areas represent upper and lower 95% CIs. Adjustment factors are as same as which presented in extended model of Table 3.

In all patients, after adjusting for sex, age, BMI, hypertension, diabetes, cardiovascular diseases, smoking, WBC, NLR, and Clavien-Dindo grade, the non-linear dose-response risk curve showed that the risk of LOS >2 weeks increased with the increase of POBG (Figure 1, Table 2). In the subgroups of controlled BMI, cardiovascular disease, and smoking, the LOS of the group (POBG ≥ 6.88 mmol/L) was significantly increased. Although there was no statistical difference in the other subgroups, the overall trend was that the length of hospital stay in the hyperglycemia group was increased.

**Table 2:** Weighted odds ratio and 95% confidence intervals of LOS > 2 weeks by levels of POBG levels.

**LOS > 2 weeks**	**5 mmol/L**	**6 mmol/L**	**7 mmol/L**	**8 mmol/L**	**9 mmol/L**	**10 mmol/L**
Overall	0.85 (0.64–1.12)	1.00 (0.90–1.11)	1.00 (0.63–1.61)	1.10 (0.58–2.08)	1.28 (0.65–2.54)	1.57 (0.76–3.23)
Male	0.90 (0.67–1.20)	1.00 (0.84–1.18)	0.96 (0.51–1.81)	1.00 (0.43–2.33)	1.11 (0.45–2.71)	1.26 (0.51–3.14)
Age ≥ 55 years	0.90 (0.56–1.46)	0.98 (0.96–1.01)	1.43 (0.87–2.34)	1.84 (0.85–3.99)	2.12 (0.88–5.10)	2.26 (0.91–5.64)
BMI ≥ 24 kg/m^2^	0.90 (0.57–1.43)	1.05 (0.93–1.19)	1.32 (0.70–2.45)	1.63 (0.67–3.94)	2.02 (0.76–5.33)	2.49 (0.92–6.73)
No hypertension	0.90 (0.64–1.29)	0.81 (0.58–1.12)	0.70 (0.33–1.50)	1.07(0.45–2.55)	2.15 (0.64–7.26)	
No diabetes	0.89 (0.66–1.19)	0.92 (0.79–1.08)	0.76 (0.44–1.32)	0.79 (0.40–1.57)	0.97 (0.47–1.99)	1.25 (0.55–2.85)
No cardiovascular diseases	0.89 (0.67–1.18)	1.01 (0.89–1.14)	1.04 (0.64–1.71)	1.17 (0.62–2.21)	1.37 (0.70–2.66)	1.64 (0.79–3.40)
No smoking	0.83 (0.63–1.10)	1.00 (0.89–1.13)	1.00 (0.61–1.63)	1.09 (0.56–2.09)	1.26 (0.62–2.55)	1.52 (0.72–3.20)

*The following covariates were adjusted: gender, age, BMI, hypertension, diabetes, cardiovascular diseases, smoking, WBC, NLR, and Clavien-Dindo grade*.

*BMI, Body mass index; LOS, Length of stay*.

**Figure 2:**
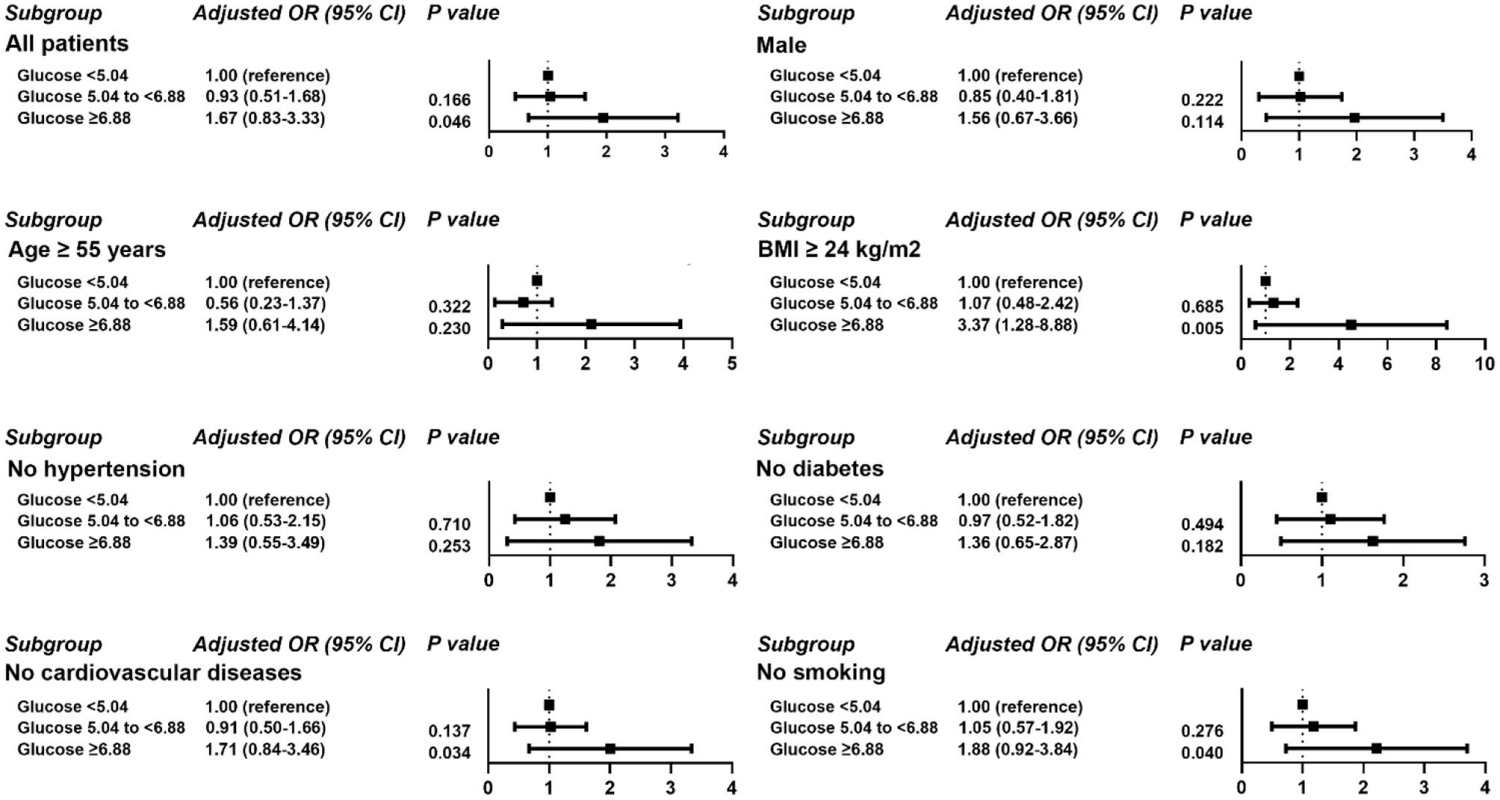
Results of subgroup analyses of POBG and a hospital length of stay > 2 weeks based on clinical characteristics.

Logistic regression was used to assess the association of POBG with hospital LOS > 2 weeks. We found that POBG was an independent risk factor for LOS > 2 weeks in the univariate analysis, the basic model, the core model, and the extended model. In the extended model, patients with POBG levels ≥ 6.88 mmol/L had a 67% higher risk of LOS > 2 weeks compared to patients with POBG levels <5.04 mmol/L (aOR 1.67; 95% CI 0.83-3.33; *P* = 0.046) (Table 3 Extended model). In addition, similar results were observed in most subgroup analyses (Figure 2).

**Table 3:** Relative risk of having a hospital LOS of > 2 weeks was calculated according to POBG level in tertile groups^a^.

**Characteristic**	***N***	**Univariate analysis**		**Basic model**		**Core model**		**Extended model**	
		**aOR (95% CI)**	***P*-value**	**aOR (95% CI)**	***P*-value**	**aOR (95% CI)**	***P*-value**	**aOR (95% CI)**	***P*-value**
**Glucose, mmol/L**
Glucose <5.04	76	1.00		1.00		1.00			
Glucose 5.04 to <6.88	156	1.16 (0.66–2.03)	0.610	0.99 (0.55–1.76)	0.146	0.96 (0.54–1.73)	0.160	1.00	
Glucose ≥6.88	78	**2.33 (1.22–4.45)**	**0.010**	**1.91 (0.97–3.74)**	**0.017**	**1.81 (0.91–3.58)**	**0.027**	0.93 (0.51–1.68)	0.166

a *Adjusted covariates: Basic model: age, gender and BMI; Core model: gender, age, BMI, hypertension, diabetes, cardiovascular diseases and smoking; Extended model: gender, age, BMI, hypertension, diabetes, cardiovascular diseases, smoking, WBC, NLR, and Clavien-Dindo grade*.

*BMI, Body mass index; LOS, Length of stay; CI, confidence interval. aOR, adjusted odds ratio. The bold values indicates p-value is less than 0.05*.

## Discussion

This retrospective study demonstrated that in patients who underwent PCNL, elevated POBG levels impacted prolonged hospital LOS, which were significantly correlated in a non-linear dose-response manner. When POBG levels were ≥6.88 mmol/L, patients had a 67% higher risk of hospital LOS> 2 weeks. Our findings provide preliminary recommendation for optimizing preoperative glycemic control.

A growing number of studies have shown that abnormal glucose regulation is associated with poor postoperative outcomes. A large retrospective cohort study showed a 19% increased risk of hospital LOS >3 days for appendectomy with POBG levels ≥6.83 mmol/L and a 17% increased risk of prolonged hospital LOS for cholecystectomy with POBG levels ≥7.11 mmol/L (19). A similar study was conducted by Davis et al. in neurosurgery patients, and they found that the POBG levels were significantly associated with longer postoperative hospital stays when the POBG level>6.67 mmol/L (20). A clinical study with a large sample explored the impact of preoperative glucose management on clinical outcomes in elective surgery. It showed that fasting glucose <11.11 mmol/L may be beneficial in reducing hospital LOS for diabetic patients, although the clinical significance was unclear (21).

Our study found that high POBG was associated with the prolonged hospital LOS. When considering how high POBG prolongs LOS, we found that patients in the high POBG group were older (60.72 ± 10.33 vs. 55.73 ± 11.18 and 51.22 ± 13.13; *p* < 0.001) and had a significantly higher proportion of hypertension (52.6% vs. 38.5% and 25.0%; *p* = 0.002) than the other two groups. The increase in age was significant for the prolongation of the hospital stay. Studies have shown that the number of days in the hospital is significantly higher in older patients admitted to the emergency room than in younger ones (22). Another study showed that hypertension was a risk factor for prolonged LOS in burn surgery patients (23).

Preoperative infection status and surgery duration have been found to be correlated with the occurrence of severe infection after PCN (24). In this study, we observed the correlation between POBG and inflammatory markers such as WBC, NLR, which indicated POBG may be an indicator of physiological state and influenced clinical outcomes such as hospital LOS. It is currently believed that diabetes leads to an increased incidence of postoperative infection due to the mechanisms followed. The stress response caused by anesthesia and surgery leads to elevated blood glucose, and the hyperglycemic state inhibits neovascularization and collagen aggregation, which affects wound healing (25). Diabetic patients have weakened immune system, decreased leukocyte chemotaxis, and declined resistance to external stimuli (26). Abnormal peripheral vascular dysfunction and microcirculatory function in diabetic patients affect the wound recovery (27). For diabetic patients, the relevant researches recommend maintaining preoperative and intraoperative blood glucose levels below 10 mmol/L (180 mg/ml), reduce the fractional risk of surgical infections and poor wound healing (10, 11, 28). We found an optimal value of 5.76 mmol/L for POBG, which is below the clinical level. Our findings provide preliminary recommendations for optimizing blood glucose levels.

This study has several limitations. First of all, this study is a single-center retrospective study, and further prospective studies of multiple healthcare systems are required to verify its accuracy. Second, we may have underestimated the correlation of hypertension and cardiovascular disease in the study, which could have an impact on hospital LOS. Third, the diagnoses of hypertension and diabetes are based on the results of the doctor's consultation at the time of the patient's admission, and there will be a proportion of patients who are unaware of their medical condition prior to admission. Finally, in this study, patients were grouped based on only one POBG test result, and further integration of patients' multiple POBG levels and preoperative glycated hemoglobin results is needed.

## Conclusion

In conclusion, we found that higher POBG levels were significantly associated with prolonged LOS in patients undergoing percutaneous nephrolithotomy.

## Data Availability Statement

The original contributions presented in the study are included in the article/Supplementary Material, further inquiries can be directed to the corresponding author/s.

## Author Contributions

SS, WM, ST, GZ, and MC: conception and design. GZ and MC: administrative support. SS, WM, LW, and XZ: collection and assembly of data. SS and WM: data analysis and interpretation. SS, WM, and LW: manuscript writing. All authors have approved the final draft of the manuscript for publication.

## Funding

This study was funded by Natural Science Foundation of China (82170703), Natural Science Foundation of China (82100732), Natural Science Foundation of Jiangsu Province (BK20200360), Excellent Youth Development Fund of Zhongda Hospital, SEU (2021ZDYYYQPY04), and Tai-Shan Scholar Program from Shandong Province (No. tsqn202103116).

## Conflict of Interest

The authors declare that the research was conducted in the absence of any commercial or financial relationships that could be construed as a potential conflict of interest.

## Publisher's Note

All claims expressed in this article are solely those of the authors and do not necessarily represent those of their affiliated organizations, or those of the publisher, the editors and the reviewers. Any product that may be evaluated in this article, or claim that may be made by its manufacturer, is not guaranteed or endorsed by the publisher.

## Supplementary Material

Supplementary Figure 1 Relationship between White blood cell (WBC) count and POBG (Left). Relationship between with neutrophil-to-lymphocyte ratio (NLR) and POBG (Right).
