# Supplementary figures and images for: Association Between Preoperative Blood Glucose Level and Hospital Length of Stay in Patients With Kidney Stones Undergoing Percutaneous Nephrolithotomy
Source: Front Surg. 2022 Jan 20;8:820018. doi: 10.3389/fsurg.2021.820018 (PMC8811039; doi:10.3389/fsurg.2021.820018)

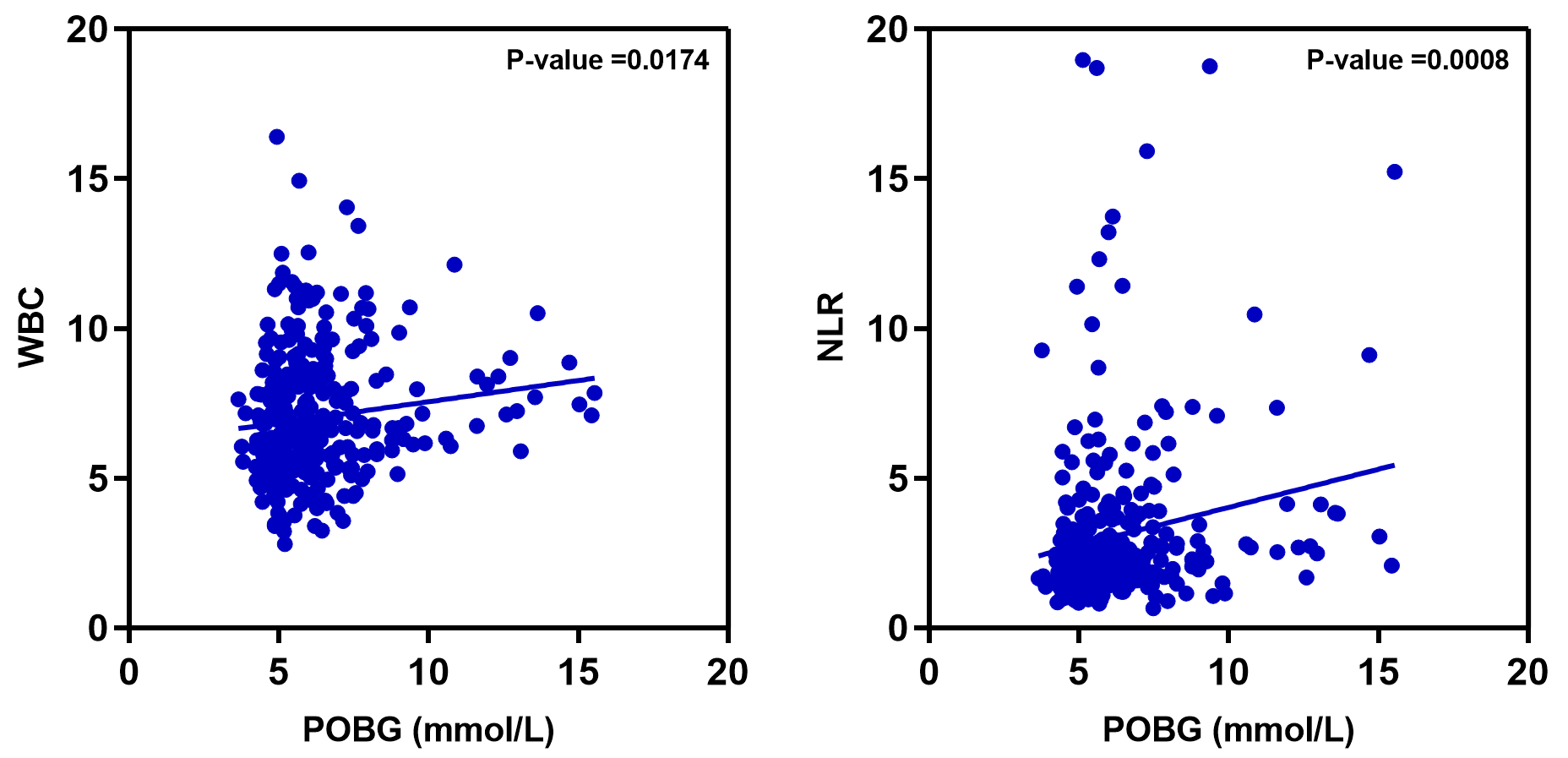

Supplement: Supplementary Figure 1 — Relationship between White blood cell (WBC) count and POBG (Left). Relationship between with neutrophil-to-lymphocyte ratio (NLR) and POBG (Right). [file Image_1.tif]
